# Complete chloroplast genome sequence of *Ilex latifolia* (Aquifoliaceae), a traditional Chinese tea

**DOI:** 10.1080/23802359.2019.1698985

**Published:** 2019-12-13

**Authors:** Yancai Shi, Bingbing Liu

**Affiliations:** a Institute of Loess Plateau, Shanxi University, Taiyuan, China;; b Guangxi Institute of Botany, Guangxi Zhuang Autonomous Region and Chinese Academy of Sciences, Guilin, China

**Keywords:** Ilex, chloroplast genome, phylogenetic analysis

## Abstract

*Ilex latifolia* (Aquifoliaceae), commonly known as ‘kudingcha’, is an evergreen tree widely distributed in southwest China. It is well known for its health function due to possess antioxidant, antidiabetic, antihypertensive, anti-inflammatory, and anti-ischemic activities. Here, we first report and characterize its complete chloroplast genome based on Illumina paired-end sequencing data. The complete plastid genome was 157,601 bp, which contained inverted repeats (IR) of 26,077 bp separated by a large single-copy (LSC) and a small single copy (SSC) of 87,020 bp and 18,427 bp, respectively. The cpDNA contains 143 genes, comprising 95 protein-coding genes, 40 tRNA genes, and eight rRNA genes. The overall GC content of the plastome is 37.6%. The phylogenetic analysis of 18 selected chloroplast genomes demonstrated that *I. latifolia* is closely related to the congeneric *I*. *integra*.

*Ilex latifolia* (Thunb.), which belongs to the *Ilex* genus in Aquifoliaceae family, is an evergreen tree widely distributed in southwest China. In China, *I. latifolia*, commonly known as ‘kudingcha’, is a particularly bitter-tasting tea that has been widely used for almost 2000 years. It is well known for its health function due to possess antioxidant, antidiabetic, antihypertensive, anti-inflammatory, and anti-ischemic activities (Nishimura et al. 1999). However, genetic and genomic resource of the species is very limited. Herein, we first report and characterize its complete plastome based on Illumina paired-end sequencing data, which will contribute to the further studies on its genetic research and resource utilization. The annotated cp genome of *I. latifolia* has been deposited into GenBank with the accession number MN688228.

In this study, *I. latifolia* was sampled from in Guangxi Zhuang Autonomous Region of China, located at 103°47′22″E, 22°17′22″N. A voucher specimen (Y.-C. Shi et al. H1127) was deposited in the Guangxi Key Laboratory of Plant Conservation and Restoration Ecology in Karst Terrain, Guangxi Institute of Botany, Guangxi Zhuang Autonomous Region and Chinese Academy of Sciences, Guilin, China. The experiment procedure is as reported in Zhang et al. (2019). Around 2 Gb clean data were used for the cp genome de novo assembly by the program NOVOPlasty (Dierckxsens et al. 2017) and direct-viewing in Geneious R11 (Biomatters Ltd., Auckland, New Zealand). Annotation was performed with the program Plann (Huang and Cronk 2015) and Sequin (http://www.ncbi.nlm.nih.gov/).

The chloroplast genome of *I. latifolia* is a typical quadripartite structure with a length of 157,601 bp, which contained inverted repeats (IR) of 26,077 bp separated by a large single-copy (LSC) and a small single copy (SSC) of 87,020 bp and 18,427 bp, respectively. The cpDNA contains 143 genes, comprising 95 protein-coding genes, 40 tRNA genes, and eight rRNA genes. Among the annotated genes, 13 of them contain one intron (*atp*F, *ndh*A, *ndh*B, *rps*12, *rpoC*1, *pet*B, *rpl*2, *trn*A-UGC, *trn*I-GAU, *trn*G-GCC, *trn*K-UUU, *trn*L-UAA, and *trn*V-UAC), and two genes (*clp*P and *ycf*3) contain two introns. The overall GC content of the plastome is 37.6%.

To identify the phylogenetic position of *I. latifolia*, phylogenetic analysis was conducted. A neighbor-joining (NJ) tree with 1000 bootstrap replicates was inferred using MEGA version 7 (Kumar et al. 2016) from alignments created using the MAFFT (Katoh and Standley 2013) using plastid genomes of 17 species. It showed the position of *I. latifolia* was close to the congeneric *I*. *integra* (Figure 1). Our findings can be further used for plastome evolution, population genomic and phylogenomic studies of Aquifoliaceae. It will also provide fundamental data for the utilization and management of this important medicinal plant.

**Figure 1.**
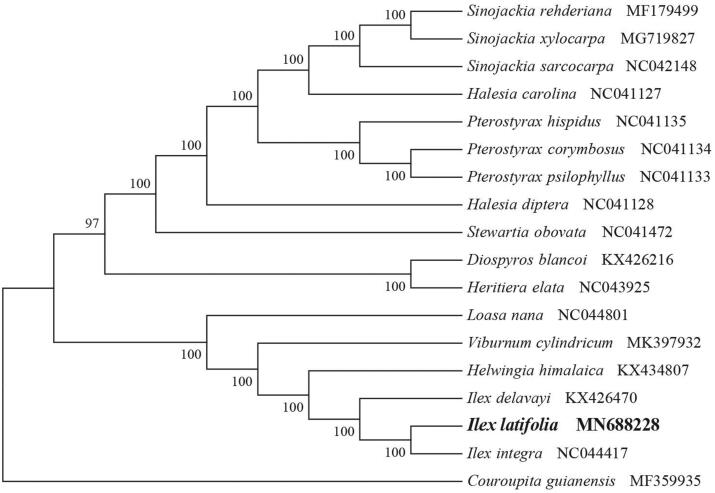
NJ phylogenetic tree of *I. latifolia* with 17 species was constructed by chloroplast plastome sequences. Numbers on the nodes are bootstrap values from 1000 replicates. *Couroupita guianensis* was selected as outgroup.

## References

[CIT0001] Dierckxsens N, Mardulyn P, Smits G. 2017. NOVOPlasty: de novo assembly of organelle genomes from whole genome data. Nucleic Acids Res. 45(4):e18. 28204566 10.1093/nar/gkw955 PMC5389512

[CIT0002] Huang DI, Cronk Q. 2015. Plann: a command-line application for annotating plastome sequences. Appl Plant Sci. 3(8):1500026. 10.3732/apps.1500026 PMC4542940 26312193

[CIT0003] Katoh K, Standley DM. 2013. MAFFT multiple sequence alignment software version 7: improvements in performance and usability. Mol Biol Evol. 30(4):772–780. 23329690 10.1093/molbev/mst010 PMC3603318

[CIT0004] Kumar S, Stecher G, Tamura K. 2016. MEGA7: molecular evolutionary genetics analysis version 7.0 for bigger datasets. Mol Biol Evol. 33(7):1870–1874. 27004904 10.1093/molbev/msw054 PMC8210823

[CIT0005] Nishimura K, Fukuda T, Miyase T, Noguchi H, Chen XM. 1999. Activity-guided isolation of triterpenoid acyl CoA cholesteryl acyl transferase (ACAT) inhibitors from *Ilex kudincha*. J Nat Prod. 62(7):1061–1064. 10425145 10.1021/np990019j

[CIT0006] Zhang Y, Shi YC, Duan N, Liu BB, Mi J. 2019. Complete chloroplast genome of *Euphorbia tirucalli* (Euphorbiaceae), a potential biofuel plant. Mitochondrial DNA B. 4(1):1973–1974.

